# Fit for consumption: zebrafish as a model for tuberculosis

**DOI:** 10.1242/dmm.016089

**Published:** 2014-07

**Authors:** Mark R. Cronan, David M. Tobin

**Affiliations:** 1 Department of Molecular Genetics and Microbiology, Duke University Medical Center, Durham, NC 27710, USA.; 2 Center for Microbial Pathogenesis, Duke University Medical Center, Durham, NC 27710, USA.

**Keywords:** Disease models, Genetics, Mycobacterium, Pathogenesis, Tuberculosis, Zebrafish

## Abstract

Despite efforts to generate new vaccines and antibiotics for tuberculosis, the disease remains a public health problem worldwide. The zebrafish *Danio rerio* has emerged as a useful model to investigate mycobacterial pathogenesis and treatment. Infection of zebrafish with *Mycobacterium marinum*, the closest relative of the *Mycobacterium tuberculosis* complex, recapitulates many aspects of human tuberculosis. The zebrafish model affords optical transparency, abundant genetic tools and *in vivo* imaging of the progression of infection. Here, we review how the zebrafish–*M. marinum* system has been deployed to make novel observations about the role of innate immunity, the tuberculous granuloma, and crucial host and bacterial genes. Finally, we assess how these findings relate to human disease and provide a framework for novel strategies to treat tuberculosis.

## Introduction

### Tuberculosis pathology in humans

Since arising as a human pathogen an estimated 70,000 years ago, *Mycobacterium tuberculosis* (*Mtb*) has been a major cause of mortality and morbidity in human populations (Comas et al., 2013). Although *Mtb* was identified as the cause of tuberculosis (TB) in the 1880s, current therapeutic options have not been able to eradicate the disease. More than 8 million cases of TB were diagnosed in 2012, leading to 1.3 million deaths worldwide (WHO, 2013).

Clinically, the ongoing mortality of TB results in part from the difficulty in treating *Mtb* with existing antibiotics. Current frontline antibiotic combinations require months-long treatment courses, making treatment difficult, particularly in the developing world where access to healthcare can be limited (Sacchettini et al., 2008). This high rate of treatment failure has led to the appearance of multidrug-resistant (MDR) and extremely drug-resistant (XDR) strains, which further contribute to clinical burden and mortality (Sacchettini et al., 2008). Furthermore, although a number of promising new vaccine approaches have been described in recent years, the *Mycobacterium bovis* bacillus Calmette–Guérin (BCG) vaccine, in use for almost a century, remains the only vaccine deployed throughout the world (Ottenhoff and Kaufmann, 2012). BCG vaccination protects from disseminated TB, particularly TB meningitis in younger patients, but has little efficacy in adult populations (Ottenhoff and Kaufmann, 2012). Thus, although vaccine and treatment options exist for TB, their suboptimal performance contributes to the continued public health burden of tuberculosis. To address the limited efficacy of existing treatment options, ongoing research has sought to better understand the process of *Mtb* pathogenesis, and to identify novel host and bacterial genes that are crucial for *Mtb* infection.

During *Mtb* infection, the bacterium is taken up by host phagocytes and replicates within them (Wolf et al., 2007; Eum et al., 2010; Repasy et al., 2013). As infection progresses, macrophages and other immune cell types are recruited to the lung and other sites of infection, creating a structured aggregate of infected and uninfected cells called the granuloma (Philips and Ernst, 2012; Ramakrishnan, 2012; Russell, 2013). These granulomas consist of a tightly interdigitated inner core of macrophages termed ‘epithelioid’ macrophages, surrounded by additional immune cells, including T-cells, B-cells, dendritic cells and neutrophils (Philips and Ernst, 2012; Ramakrishnan, 2012; Russell, 2013). The inner core of these granulomas undergoes pronounced changes, including the induction of necrosis and hypoxia, resulting in a central core filled with lipid-rich cell debris termed caseum (Philips and Ernst, 2012; Ramakrishnan, 2012; Russell, 2013). The granuloma is the hallmark structure of tuberculosis and constitutes a crucial niche in which bacteria persist. Eventual rupture of granulomas is crucial to bacterial release into the lung and transmission of the disease.

### Modeling Mtb infection *in vitro* and in animal models

A number of groups have used *Mtb* cultures *in vitro* or *Mtb*-infected macrophages to rapidly identify host and bacterial genes that constitute targets for novel anti-tubercular therapeutics (Sassetti et al., 2001; Sassetti et al., 2003; Jayaswal et al., 2010; Kumar et al., 2010). However, translating these results into patients is limited by major differences between broth-grown *Mtb*, cultured macrophages infected with *Mtb* and the *in vivo* reality of *Mtb* growth within the complex architecture of the granuloma. To these ends, groups have generated elegant mixed cell culture models that can give rise to granuloma-like structures *in vitro* (Puissegur et al., 2004; Kapoor et al., 2013). However, validation of host and bacterial genes involved in infection and novel TB drugs still requires the use of animal models of tuberculosis infection. Thus, progress in TB research has been linked to the development of animal models that can functionally reproduce the pathology and bacterial growth characteristics observed in humans.

Of the many animal models developed for *Mtb* infection, each has distinct strengths and weaknesses. The most popular has been the mouse infection model. The mouse model is particularly versatile because of the extensive range of tools and strains available for mice. However, it can be limited in its ability to reproduce the observed human pathologies (Flynn, 2006). Rather than the compact, caseating granulomas observed in humans, the most widely used mouse models (C57BL6 and BALB/c) form granulomas that are comparatively diffuse and fail to caseate (Flynn, 2006). Newer mouse models, including the C3HeB/FeJ ‘Kramnik’ model, *Nos2*^−/−^ C57BL/6 mice and *IL10*^−/−^ CBA/J mice, form granulomas that undergo caseating necrosis (Kramnik et al., 2000; Reece et al., 2010; Cyktor et al., 2013), but use of these models with existing knockout lines requires extensive crossing. In addition to mouse models, researchers have adopted other mammalian models that closely mimic human TB pathology, including guinea pigs and rabbits, which form necrotic granulomas (Flynn, 2006). However, the rabbit and guinea pig models lack the catalog of existing reagents available for mice, and are not nearly as amenable for transgenic and knockout line production as mice. The most clinically relevant model has been the primate infection model (Capuano et al., 2003). Nonetheless, the cost, time and ethical considerations of this model mean that it can only be used in a limited fashion. Thus, researchers continue to search for models of *Mtb* infection that can offer new insights into *Mtb* pathogenesis. The emergence of a zebrafish mycobacterial infection model, using the closely related mycobacterial pathogen *M. marinum*, complements these models. It recapitulates many aspects of human tuberculosis infection, and its unique characteristics have expanded experimental possibilities, leading to new insights about mycobacterial pathogenesis that have been validated in human disease.

## The zebrafish model of mycobacterial infection

**Fig. 1.**
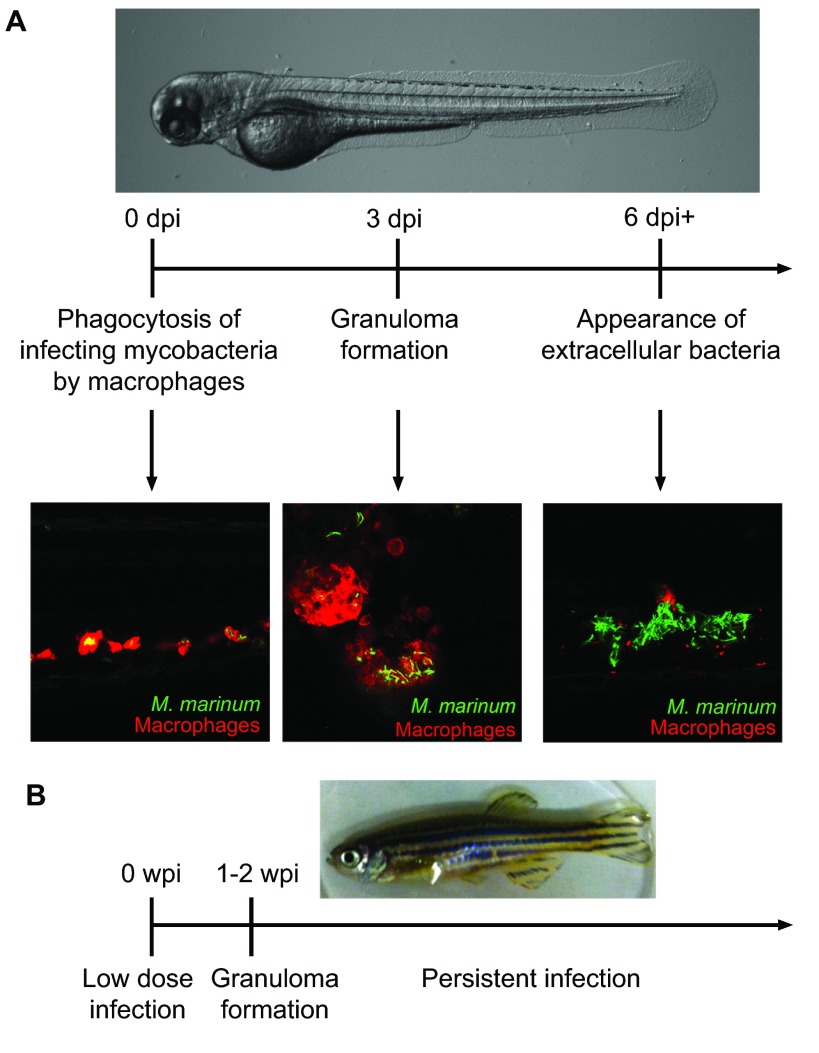
Modeling mycobacterial infection in larval and adult zebrafish. (A) From top – brightfield image of a zebrafish larva. Middle – an approximate timeline of progression of infection in zebrafish larvae, in days post-infection (dpi). Bottom – confocal images of zebrafish lines, with fluorescently labeled macrophages shown in red and infecting fluorescent mycobacteria visualized in green. Representative images display from left – scattered infected macrophages; center – macrophages aggregated into granulomas; right – the appearance of extracellular bacteria as containment fails at isolated granulomas. (B) Top – image of an adult zebrafish. Bottom – a timeline of infection progression in weeks post-infection (wpi) in the adult persistent infection model (based on the work of Parikka et al., 2012).

The zebrafish immune system has extensive homology with the human immune system, possessing both innate and adaptive arms (Renshaw and Trede, 2012). Systemic infection of zebrafish with *M. marinum* results in phagocytosis of infecting bacteria by macrophages (Fig. 1A) and formation of caseating granulomas that are histologically similar to human *Mtb* granulomas (Davis et al., 2002; Swaim et al., 2006). Functionally, crucial virulence factors, host genes and immune cell types implicated in human *Mtb* pathogenesis have conserved functions within the zebrafish*–M. marinum* model (Gao et al., 2004; Volkman et al., 2004; Cosma et al., 2006; Swaim et al., 2006; Clay et al., 2008; Stoop et al., 2011; van der Woude et al., 2012; Kanwal et al., 2013; Stoop et al., 2013; van der Vaart et al., 2013).

The similarities between human and zebrafish mycobacterial infection are striking, and the zebrafish*–M. marinum* model presents additional advantages that are distinct from those of other mycobacterial infection models. First, using optically clear zebrafish larvae, researchers are able to directly observe the process of active infection in real time (Fig. 1A) (Davis et al., 2002). Second, zebrafish larvae possess only the innate immune system, but still show robust granuloma formation (Fig. 1A) (Davis et al., 2002). Thus, infection of zebrafish larvae enables researchers to study relevant pathology using a subgroup of cell types. Third, both host and bacteria are genetically tractable and easy to obtain in large numbers. Zebrafish transgenesis methods have been well characterized, and the recent breakthroughs in gene editing with transcription-activator-like effector nucleases (TALENs) and CRISPR/Cas9 have enabled rapid and facile gene knockouts in zebrafish (Kawakami et al., 2004; Huang et al., 2011; Sander et al., 2011; Hwang et al., 2013). On the bacterial side, the rapid generation time (4 hours for *M. marinum* versus ~20–24 hours for *Mtb*) facilitates construction of knockout and transposon-containing strains, and results in an infection model in which experiments are conducted within the time frame of days to weeks rather than weeks to months. Furthermore, zebrafish have been experimentally infected with a wide range of bacterial pathogens, allowing comparison of mycobacterial infection phenotypes with other pathogenic bacteria (Meijer and Spaink, 2011; Torraca et al., 2014). Finally, in conjunction with facile transgenesis and optical clarity, a number of promoters specific for distinct immune lineages have recently been described in zebrafish, enabling direct observation of these cell types by fluorescent protein expression and interrogation of gene function by cell-type-specific expression constructs (Langenau et al., 2004; Renshaw et al., 2006; Hall et al., 2007; Ellett et al., 2011; Torraca et al., 2014). Beyond the larvae, adult zebrafish infections have enabled researchers to investigate mycobacterial infection in the context of both the innate and adaptive immune systems. Low-dose infection of adult zebrafish with the Aronson strain of *M. marinum* leads to persistent infections resembling latency that can be reactivated by immunocompromise (Fig. 1B) (Parikka et al., 2012). In the following sections, we will discuss how researchers taking advantage of the unique benefits of the zebrafish–*M. marinum* system have been able to answer longstanding questions and open new fields of inquiry regarding innate immunity, granuloma dynamics, and host and bacterial genetics within mycobacterial pathogenesis.

## Innate immunity

As an early host cell of mycobacteria, macrophages play a central role in mycobacterial pathogenesis and granuloma formation. Despite the well-known phagocytic capacity of macrophages, mycobacteria readily persist within these cells (Clay et al., 2007). Macrophages do serve to restrict proliferation of these persistent mycobacteria; extracellular *M. marinum* grow rapidly in zebrafish specifically depleted of macrophages (Clay et al., 2007). However, studies in macrophage-depleted zebrafish also indicate that mycobacteria co-opt this highly motile cell type to seed new infection foci in surrounding tissues within the host (Clay et al., 2007).

**Fig. 2.**
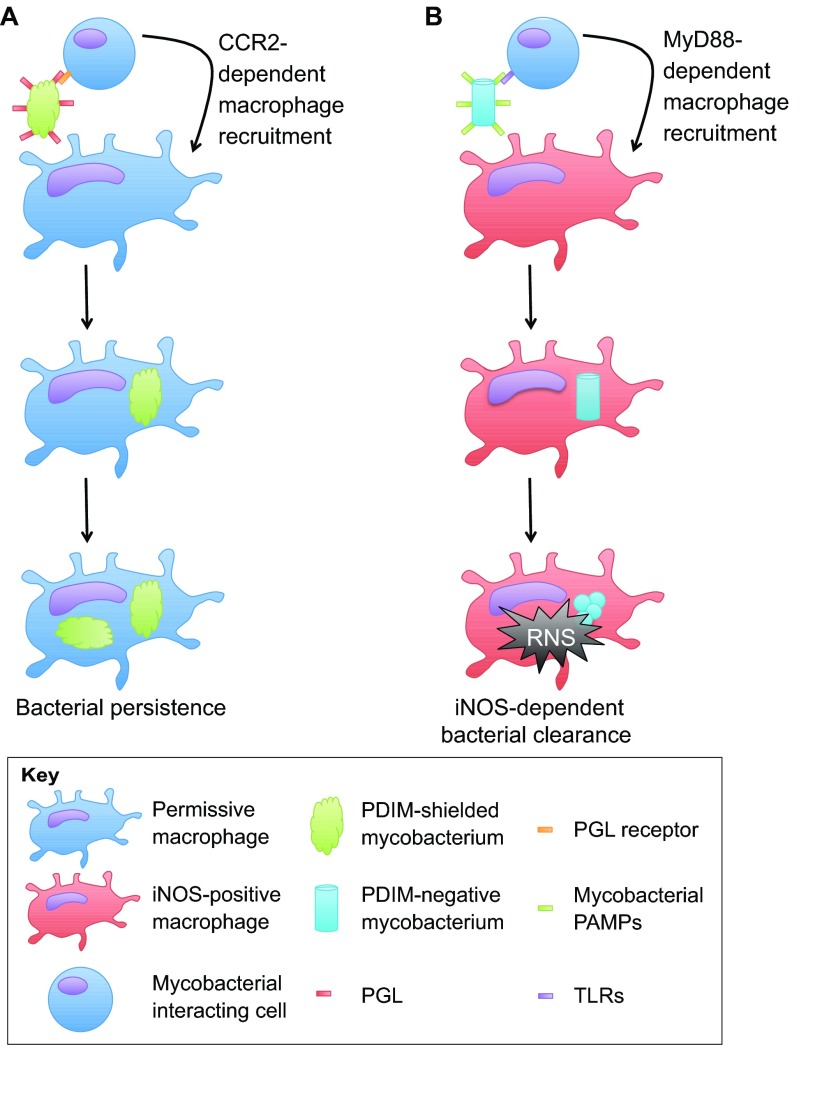
**Mycobacterial lipids facilitate bacterial uptake into permissive macrophage populations.** (A) PDIM shields wild-type mycobacteria (green) from TLR-mediated recognition of mycobacteria by the host cell. Interaction of PGL on the bacterial cell surface with surrounding host cells drives CCR2-mediated recruitment of mycobacterial permissive macrophages. These permissive macrophages phagocytose the mycobacterium, which can subsequently form a persistent infection within the host. (B) In PDIM-deficient mycobacteria (light blue), bacterial PAMPs are recognized by TLRs, leading to MyD88-dependent recruitment of iNOS-positive macrophages. These iNOS-positive macrophages phagocytose the infecting mycobacteria and rapidly clear them through iNOS-dependent RNS.

Researchers have sought to understand how mycobacteria persist within a professional phagocyte. Findings in the zebrafish–*M. marinum* model suggest that mycobacteria promote their persistence by selectively enhancing phagocytosis into subsets of macrophages deficient for inducible nitric oxide synthase (iNOS), which have reduced microbicidal activity (Cambier et al., 2014). Using mycobacteria lacking cell-wall components phthiocerol dimycoceroserate (PDIM) and phenolic glycolipids (PGLs), two lipid species previously found to be involved in *Mtb* virulence (Cox et al., 1999; Reed et al., 2004), and zebrafish depleted for the toll-like receptor (TLR) adaptor MyD88, the mechanistic underpinnings of this selective trafficking have been mapped *in vivo* (Cambier et al., 2014). PDIM served to hide mycobacteria from pattern-recognition receptors, avoiding TLR-dependent recognition of mycobacterial pathogen-associated molecular patterns (PAMPs), macrophage recruitment, phagocytosis by iNOS-positive macrophages and reactive nitrogen species (RNS)-mediated killing (Cambier et al., 2014). PGL, by contrast, recruited iNOS-deficient macrophages to bacteria in a manner dependent upon the chemokine receptor CCR2 (Fig. 2) (Cambier et al., 2014). In this and other studies, *M. marinum* deficient in PDIM and/or PGL were attenuated in zebrafish (Alibaud et al., 2011; Yu et al., 2012; Cambier et al., 2014). These findings indicate that mycobacterial phagocytosis, rather than being exclusively host driven, is instead a process tuned by the mycobacteria themselves to establish a productive niche for their growth and dissemination.

The macrophage is a major cell type recruited to and infected by *Mtb*; however, other immune cell types are also infected at high rates *in vivo*, including neutrophils (Wolf et al., 2007; Eum et al., 2010; Repasy et al., 2013). In mammalian models, studies have suggested that neutrophils probably play a protective role in *Mtb* infection (Lowe et al., 2012). However, *ex vivo* studies of mycobacterial killing by neutrophils have been contentious, probably because of the short half-life of this cell type and its sensitivity to conditions during isolation from blood (Lowe et al., 2012). Transgenic zebrafish in which both neutrophils and mycobacteria are fluorescently tagged have enabled direct interrogation of the interactions between neutrophils and mycobacteria *in vivo* (Meijer et al., 2008; Yang et al., 2012). In contrast to cell culture studies, in which neutrophils readily phagocytose mycobacteria, *in vivo* neutrophils fail to phagocytose free bacteria (Jones et al., 1990; Majeed et al., 1998; Yang et al., 2012). Instead, neutrophils become infected upon recruitment to established granulomas, where they phagocytose dead and dying infected macrophages (Yang et al., 2012). Previous work in zebrafish has demonstrated that macrophages readily phagocytose bacteria in fluid environments, whereas neutrophils can only phagocytose surface-associated microbes (Colucci-Guyon et al., 2011). Preferential uptake of mycobacteria into macrophages in this model might be due to the venous route of infection used in this model (Yang et al., 2012). However, in natural *Mtb* infections, alveolar macrophages are thought to be the first cells to encounter and phagocytose *Mtb* (Philips and Ernst, 2012). Consistent with initial infection of alveolar macrophages, in experimental mouse infections it was observed that *Mtb* solely infects monocytic cells at the earliest time points (Repasy et al., 2013). Thus, venous delivery of mycobacteria in zebrafish seems to mimic human *Mtb* infection, whereby neutrophils encounter mycobacteria only after transit through alveolar macrophages.

Upon uptake of infected cell debris, a subset of motile neutrophils actively kills the phagocytosed mycobacteria by means of oxidative mechanisms (Yang et al., 2012). Oxidative killing of mycobacteria is consistent with another study in which signaling through the transcription factor hypoxia-inducible factor 1-alpha (Hif-1α) enhances production of RNS by neutrophils and hence mycobacterial killing (Elks et al., 2013). To assess functionally the contribution of neutrophil recruitment and subsequent mycobacterial killing to pathogenesis, Yang and colleagues took advantage of a zebrafish line in which neutrophil recruitment was inhibited by expression of a truncated form of the chemokine receptor CXCR4b in neutrophils. In this mutant line, the failure of neutrophil recruitment to granulomas resulted in an enhanced mycobacterial burden within infected animals (Yang et al., 2012). These results indicate that innate immune cell types other than macrophages also contribute to mycobacterial pathogenesis. The recent discovery of zebrafish dendritic cells, mast cells and eosinophils suggests that the zebrafish model could be used to help clarify the involvement of other innate immune cell types during mycobacterial infection (Lieschke et al., 2001; Dobson et al., 2008; Balla et al., 2010; Lugo-Villarino et al., 2010; Wittamer et al., 2011).

During the recruitment of innate immune cells, the balance of pro-and anti-inflammatory molecules controls innate immune function. Dysregulation of this inflammatory milieu can render the host susceptible to infection. Depletion of the tyrosine-protein phosphatase Ptpn6 leads to hyperinflammation in zebrafish and exacerbated infection with both *M. marinum* and *Salmonella typhimurium* (Kanwal et al., 2013). Transcriptional profiling of Ptpn6-depleted animals showed elevated levels of pro-inflammatory cytokines and pathogen-recognition molecules that were further potentiated by infection. These findings illustrate the importance of a balanced inflammatory state during mycobacterial pathogenesis.

Orthologs of macrophage-derived and mycobacteria-relevant cytokines, such as the interleukins IL-4, IL-6, IL-10, IL-12 and IL-13, plus interferon-gamma (IFN-γ) and tumor necrosis factor (TNF), have been identified in zebrafish (Zhang et al., 2005; Igawa et al., 2006; Clay et al., 2008; Ohtani et al., 2008; Holt et al., 2011; Varela et al., 2012). Only TNF, a cytokine that has long been recognized as a crucial host-protective factor in *Mtb* infection, has been extensively studied in the zebrafish–*M. marinum* model (Clay et al., 2008; Roca and Ramakrishnan, 2013). Experiments in the zebrafish demonstrated the significance of a balanced TNF response to *Mtb* pathogenesis. Induction of a hypoinflammatory, low-TNF signaling, state through morpholino knockdown of the TNF receptor led to enhanced mycobacterial growth and loss of bacterial containment through necrosis of highly infected macrophages (Clay et al., 2008). These findings are in agreement with findings in monkeys and humans, in which TNF is crucial in limiting mycobacterial growth during persistent infections (Keane et al., 2001; Lin et al., 2010). However, hyperinflammation in the zebrafish due to high TNF levels was also unfavorable for the host (Tobin et al., 2012; Roca and Ramakrishnan, 2013). Longitudinal imaging demonstrated that high TNF levels initially diminish the bacterial burden through enhanced mycobacterial killing, but bacterial restriction later fails as macrophages undergo programmed necrosis induced by mitochondrial reactive oxygen species (Roca and Ramakrishnan, 2013). Ultimately, low and high TNF states were found to be similarly unfavorable to the zebrafish host, owing to the subsequent rapid growth of extracellular bacteria (Clay et al., 2008; Tobin et al., 2010; Tobin et al., 2012; Roca and Ramakrishnan, 2013). However, the distinct mechanism of failure for the high-TNF state enabled the authors to uncouple the negative effects of high TNF (macrophage cell death) from the positive effects of high TNF (mycobacterial killing) by chemical inhibition of the mitochondrial pore complex and ceramide release (Roca and Ramakrishnan, 2013). Promisingly, high-TNF animals treated with this regimen restricted mycobacteria better than either wild-type or low-TNF animals (Roca and Ramakrishnan, 2013). Overall, these results demonstrate that the mechanistic understandings gained through the use of the zebrafish model can be used to generate new rationally designed drug therapies for mycobacterial infection.

## Granuloma formation and dynamics

Two longstanding assumptions within the tuberculosis community have shaped our expectations of the granuloma – first, that the granuloma is exclusively a host-driven protective structure and second that the granuloma is a static, wall-like structure. However, taking advantage of the visual accessibility of the zebrafish–*M. marinum* model, researchers have started to find that our traditional ideas based on pathology might not tell the whole story.

The first assumption – that of the host-driven granuloma – has been tested in longitudinal imaging experiments in zebrafish. Using mycobacteria lacking the RD1 virulence locus, the bacterial RD1 locus was found to be required for efficient granuloma formation, indicating that a virulence factor works to accelerate granuloma formation (Volkman et al., 2004). RD1 deficiency also led to attenuated *M. marinum* infection, indicating that loss of RD1-mediated granuloma formation might limit mycobacterial virulence as well (Volkman et al., 2004). The RD1 region, originally identified as a crucial pathogenicity locus that is lost in the attenuated vaccine strain BCG, encompasses a type VII secretion system called ESX-1 that is conserved in *M. marinum* (Volkman et al., 2004). Further examination of *M. marinum* ESX-1 components identified multiple bacterial genes within the ESX-1 locus required for optimal granuloma formation and virulence *in vivo*, including the early secretory antigenic target ESAT-6, the ATPase EccA1 and the ESX-1 secretion-associated protein EspL (Gao et al., 2004; Volkman et al., 2010; Stoop et al., 2011; Joshi et al., 2012). Secretion systems other than the ESX-1 machinery are also crucial to mycobacterial granuloma formation. For example, *M. marinum* strains lacking SecA2 secretion were also found to have diminished granuloma formation (Watkins et al., 2012). These findings indicate that mycobacteria have evolved to use multiple secretion systems to facilitate granuloma formation.

**Fig. 3.**
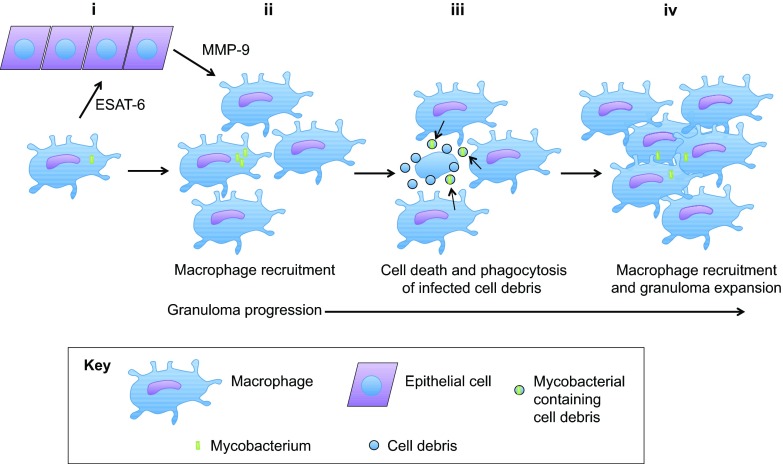
**Macrophage recruitment and granuloma formation during mycobacterial infection.** (i) ESAT-6 released from an infected macrophage drives MMP-9 production in surrounding epithelial cells. (ii) MMP-9-dependent and -independent signals drive recruitment of surrounding macrophages to the infected macrophage. (iii) The infected macrophage undergoes cell death, releasing infected cell debris. This infected cell debris is phagocytosed by recruited macrophages, expanding the population of infected macrophages. (iv) The newly infected macrophages continue to recruit additional macrophages through MMP-9-dependent and -independent mechanisms, as in panels i and ii, and ongoing cell death of infected macrophages continues to expand bacterial infection to recruited macrophage populations. As these processes continue, these infected and uninfected macrophages aggregate to form granulomas.

To better understand how mycobacterial ESX-1 secretion drives granuloma formation, individual infected macrophages were followed in zebrafish. It was found that the ESX-1 system drives cell death of infected macrophages (Volkman et al., 2004; Davis and Ramakrishnan, 2009). This locally expands the population of infected macrophages through phagocytosis of the associated mycobacterium-containing cell debris by multiple incoming macrophages (Fig. 3) (Davis and Ramakrishnan, 2009). The ESX-1 machinery also enhances recruitment of surrounding macrophages to the nascent infection (Davis and Ramakrishnan, 2009). ESX-1-dependent macrophage recruitment is driven by secretion of ESAT-6 into the surrounding tissue. Secreted ESAT-6 induces the inflammatory matrix metalloprotease MMP-9 in adjacent epithelial cells, acting as a guidance cue for nearby macrophages (Volkman et al., 2010). Successive waves of RD1-dependent macrophage cell death and recruitment further enhance bacterial expansion and granuloma formation (Fig. 3) (Davis and Ramakrishnan, 2009; Volkman et al., 2010). Granuloma formation coincides with a rapid bacterial expansion, indicating that this bacterially driven structure probably enhances this subsequent outgrowth of bacteria (Volkman et al., 2004). Thus, in contrast to the established model of an exclusively host-protective granuloma, the visual access afforded by the zebrafish–*M. marinum* model has allowed researchers to recognize that the early granuloma is also a bacterially influenced structure that can enhance mycobacterial pathogenicity. However, the granuloma might serve a dual purpose whereby, during later stages of infection, it can also exert a host-protective role.

Visualization of granulomas in zebrafish has revealed that the granuloma is a dynamic structure. Differential interference contrast (DIC) imaging of the granuloma revealed that, despite its tightly packed structure, there was considerable movement of cells into and within the granuloma (Davis et al., 2002). This was later confirmed in superinfection experiments, in which an infected animal is subsequently infected with a second strain. Using multiple fluorescent strains of *M. marinum*, it was found that superinfecting mycobacteria readily home to existing granulomas, indicating that this structure could dynamically rearrange to accommodate incoming macrophages and their phagocytosed mycobacteria (Cosma et al., 2004). Homing of superinfecting mycobacteria into established granulomas was subsequently confirmed in mouse–*Mtb* infections, demonstrating that this behavior is conserved across pathogenic mycobacteria (Cosma et al., 2008). These data, taken together with the active RD1-dependent recruitment of macrophages during infection, characterizes a dynamic granuloma within the zebrafish, at odds with the staid granulomas of medical textbooks. Researchers have also observed the emigration of infected macrophages from the granuloma (Davis and Ramakrishnan, 2009). These departing macrophages alight in other tissues, seeding new granulomas at the distal locations (Davis and Ramakrishnan, 2009). Thus, rather than a static host-protective structure, the granuloma can be dynamically remodeled to the benefit of infecting mycobacteria. In agreement with these findings from the zebrafish–*M. marinum* model, positron emission tomography imaging of lung granulomas in *Mtb*-infected macaques found that individual lesions were dynamic, freely progressing, regressing or coalescing with neighboring lesions over time (Lin et al., 2014).

## Bacterial and host genetics

### Mycobacterial factors

A dizzying array of *Mtb* virulence factors have been described in cell-culture and animal-infection models. However, reconciling virulence factor phenotypes in these distinct models and mechanistically unwinding their functions is challenging. The extensive conservation between *M. marinum* and *Mtb* virulence factors enables researchers to exploit both the faster generation time of *M. marinum* and the visual accessibility of zebrafish to interrogate virulence mutant phenotypes within weeks to months rather than months to years. Earlier in this Review, we highlighted bacterial mutants in the RD1 locus, SecA2 and EspL, for their roles in granuloma formation, and mutants in PDIM and PGL synthesis for their role in mycobacterial recognition by host macrophages. Beyond these proteins and lipids, the zebrafish–*M. marinum* model has been applied more broadly to identify other bacterial mutants that are attenuated in virulence *in vivo*. Cell-wall mutants are an important class of attenuated *Mtb* mutants. In addition to PDIM- and PGL-deficient strains, other *M. marinum* mutants with cell-wall deficiencies have been identified, such as the *iipA*, *iipB* and *erp* mutants (Cosma et al., 2006; Gao et al., 2006). These mutants have all been found to have increased sensitivity to antibiotics and are readily killed by macrophages, leading to attenuated infections in zebrafish models (Cosma et al., 2006; Gao et al., 2006). Mycobacterial strains either lacking mannose core branching of the glycolipid lipoarabinomannan (LAM) or with reduced mycolic acid synthesis were also attenuated *in vivo* (Joshi et al., 2012; Stoop et al., 2013). However, not all cell-wall mutants are hypovirulent. A transposon screen identified a cluster of genes involved in the biosynthesis of the cell-wall component lipooligosaccharide (van der Woude et al., 2012). Knockout of a member of this biosynthetic cluster, *wecE*, resulted in selective loss of one of four species of lipooligosaccharide – lipooligosaccharide-IV – leading to hypervirulence *in vivo* (van der Woude et al., 2012).

Mycobacteria possess two distinct groups of proteins called PE and PPE proteins, named for their N-terminal sequence motifs (either proline-glutamate or proline-proline-glutamate, respectively). These families are thought to participate in virulence, and large numbers of these proteins are present in both *Mtb* (169 potential coding sequences) and *M. marinum* (281 potential coding sequences) (Stinear et al., 2008). A transposon insertion in *M. marinum* disrupting the *PPE38* gene (a PPE protein that is also present in *Mtb*) led to attenuation of *M. marinum* in zebrafish. This attenuated phenotype correlated with reduced tissue pathology in animals infected with the PPE38 mutant (Dong et al., 2012). Consistent with the reduced pathology in infected zebrafish, cell-culture experiments interrogating cytokine release found that the PPE38 mutant had diminished TNF and IL-6 expression (Dong et al., 2012). More broadly, the combined function of PE and PPE proteins was addressed using an ESX-5 mutant that is defective in PE and PPE protein secretion (Weerdenburg et al., 2012). The ESX-5 mutant was found to be modestly attenuated in larval zebrafish, but highly virulent in adult zebrafish (Weerdenburg et al., 2012). Increased virulence in adult zebrafish was accompanied by increased inflammatory gene expression and granuloma formation (Weerdenburg et al., 2012). These findings indicate that PE and PPE proteins can influence virulence, and play both pro- and anti-inflammatory roles during pathogenesis.

### Host factors

On the host side, work has uncovered a number of host factors crucial for mycobacterial recognition and growth, and ongoing screening efforts continue to identify novel host loci involved in the process of infection. As a host, zebrafish are particularly amenable to screening approaches, enabling researchers to screen in an *in vivo* system. The large number of embryos obtained from a single cross, simple infection protocol, small size and aforementioned optical clarity greatly facilitate screening efforts. To further facilitate high-throughput screening, Carvalho and colleagues have designed a machine allowing automated infection of zebrafish (Carvalho et al., 2011). Infected zebrafish arrayed in 96- or 384-well plates can be readily imaged by high-content microscopy, flow cytometry or automated plate reader approaches, allowing automated detection of mycobacterial burden and other phenotypes defined by fluorescent markers (Carvalho et al., 2011; Takaki et al., 2012). Other than visual assays, changes in gene expression can be investigated at either the whole transcriptome level or in more focused subsets of genes (Hegedűs et al., 2009; Rotman et al., 2011; Kanwal et al., 2013; van der Vaart et al., 2013; Veneman et al., 2013).

Illustrating the merits of zebrafish screening efforts, a forward-genetic screen using larvae infected with *M. marinum* from 355 distinct ENU-mutagenized founders identified a novel role for Lta4h-dependent lipoxin signaling in mycobacterial pathogenesis (Tobin et al., 2010). Based on these findings, *LTA4H* variants were investigated in human patients. Genotypes associated with either high or low LTA4H levels were both found to be detrimental in disseminated TB meningitis (Tobin et al., 2010). In contrast, heterozygous patients with intermediate levels of LTA4H were protected from TB meningitis, a finding that was subsequently modeled in zebrafish (Tobin et al., 2010; Tobin et al., 2012). Dexamethasone serves as standard-of-care adjunctive therapy in TB meningitis (Thwaites et al., 2004). Combined with modeling of human *LTA4H* genotypes and dexamethasone treatment in zebrafish, further studies in human patients revealed that the survival effect of dexamethasone in TB meningitis associated strongly with the high-activity (and not the low-activity) *LTA4H* variant, suggesting that genotype-guided therapies might be useful in humans (Tobin et al., 2012). The ability to translate zebrafish genetic screens into novel clinical insights demonstrates fundamental conservation between the zebrafish–*M. marinum* model and *Mtb* infection. Additionally, these findings suggest that data from this model can be translated from ‘fish tank to bedside’, as has previously been achieved with other zebrafish models (Ablain and Zon, 2013). Complementing traditional forward-genetic screening approaches, reverse-genetic techniques have become easier in zebrafish with the description of TILLING, TALENs and CRISPR RNAs, and the growing list of existing zebrafish mutants, allowing researchers to rapidly interrogate specific host genes (Wienholds et al., 2003; Huang et al., 2011; Sander et al., 2011; Hwang et al., 2013). Thus, the host-driven response represents a promising target of antimycobacterial therapies. Additionally, the targeting of host pathways represents a valuable strategy to limit the risks associated with the emergence of treatment-resistant mycobacteria. The array of host-directed tools available for zebrafish should facilitate *in vivo* screening for potential host targets.

## Conclusions and outlook

Studies of host–pathogen interactions that give rise to the complex pathology of tuberculosis infection have long benefited from effective use of animal models. Traditional mammalian models of *Mtb* infection have provided important insights into disease, but do not pair a pathogen with its natural host and/or lack the reagents to ask the desired experimental questions. Furthermore, visual access to live animals is relatively limited in mammalian models, restricting the questions that researchers can ask effectively. In the zebrafish–*M. marinum* model, infection of zebrafish with the closest relative of the *Mtb* complex recapitulates the major pathologic feature of *Mtb* infection – the granuloma – within a genetically tractable and optically clear native host. The zebrafish–*M. marinum* model is not intended to replace the mammalian models of *Mtb* infection. However, the particular strengths of the zebrafish–*M. marinum* model enable researchers to ask questions that are difficult, impossible or simply too expensive to answer in mammalian models. These benefits have allowed researchers to gain a new understanding of mycobacterial infection, host response and pathology, and many of these findings have now been extended to mammalian models and human populations. Going forward, the clinical reality of tuberculosis requires the development of new therapeutics. Facile manipulation of host and bacterial genetics within the zebrafish–*M. marinum* system, and the ability to translate findings from the zebrafish into therapeutic strategies for patients indicate that the zebrafish–*M. marinum* model provides an excellent platform for understanding basic questions of pathogenesis and a unique resource for developing new approaches to antimycobacterial therapies.
